# Mitigating the Mistletoe Menace: Biotechnological and Smart Management Approaches

**DOI:** 10.3390/biology11111645

**Published:** 2022-11-10

**Authors:** Gaurav Mudgal, Jaspreet Kaur, Kartar Chand, Manisha Parashar, Sanjoy K. Dhar, Gajendra B. Singh, Mayank A. Gururani

**Affiliations:** 1 University Institute of Biotechnology, Chandigarh University, Mohali 140413, Punjab, India; 2 Department of Biology, College of Science, United Arab Emirates University, Al Ain 15551, United Arab Emirates

**Keywords:** mistletoe, biocontrol, transcriptome, parasitic plant, resistance, seed dispersal, frugivore, environment, drone, remote sensing

## Abstract

**Simple Summary:**

In this article, we discuss the current status of conventional management and control approaches for mistletoes, the hemiparasitic plants that have emerged as serious pests of trees in forests and commercial fruit and timber plantations. We highlight the research and implementation gaps and discuss possible newer biotechnological interventions to be made in relation to biological control agents and mistletoe-resistant tree cultivars. We also discuss the potential of smart technological alternatives that find synergies with conventional approaches.

**Abstract:**

Mistletoes have been considered a keystone resource for biodiversity, as well as a remarkable source of medicinal attributes that attract pharmacologists. Due to their hemiparasitic nature, mistletoes leach water and nutrients, including primary and secondary metabolites, through the vascular systems of their plant hosts, primarily trees. As a result of intense mistletoe infection, the hosts suffer various growth and physiological detriments, which often lead to tree mortality. Because of their easy dispersal and widespread tropism, mistletoes have become serious pests for commercial fruit and timber plantations. A variety of physical and chemical treatment methods, along with silvicultural practices, have shaped conventional mistletoe management. Others, however, have either failed to circumvent the growing range and tropism of these parasitic plants or present significant environmental and public health risks. A biocontrol approach that could sidestep these issues has never achieved full proof of concept in real-field applications. Our review discusses the downsides of conventional mistletoe control techniques and explores the possibilities of biotechnological approaches using biocontrol agents and transgenic technologies. It is possible that smart management options will pave the way for technologically advanced solutions to mitigate mistletoes that are yet to be exploited.

## 1. Introduction

Plants have been the principal source of food and energy for almost all living beings [1,2]. Economists and agriculturalists worldwide are concerned about food security due to growing populations and concomitant reductions in agriculturally cultivable land [3]. A variety of obstacles hamper efforts to improve food production, including human encroachment on land [3] and natural disturbances (forest fires, landslides, earthquakes, floods, etc.) [4,5]. In addition to the population-expansion-driven scarcity of agriculturally cultivable areas and vanishing forest ranges, biotic and abiotic stresses are equally serious and limiting factors for agricultural production [6,7]. Abiotic stresses include drought, temperature, cold, soil infertility, salinity, and the growing environmental concern regarding pollutants such as microplastics [8]. Biotic factors include overgrazing by higher animals, diseases from viruses, fungi, and bacteria, insect infestations, weeds, and parasitic plants. Most of these stressors, however, only episodically affect the growth of plants [9]. In contrast, parasitic plants such as mistletoes exhibit a prolonged presence, with many equating their senescence to that of their hosts [10,11,12,13,14,15,16]. 

There are nearly 5000 parasitic plant species belonging to 20 families of angiosperms, and many of them cause significant losses in crops used for food and industrial purposes [17,18]. Alongside the world’s major plant pests, *Striga*, *Orobanche*, *Cuscuta*, and mistletoes are more pronounced [16,19,20,21,22,23,24,25]. *Striga*, *Orobanche,* and *Cuscuta* have been reviewed extensively [19,20,21,22,23,24], but we would like to call attention to mistletoe, which has emerged as a growing global problem and requires better management and control [26]. 

The mistletoe is a hemiparasitic plant that clings to trees and depletes them of nutrients and materials, and, in many cases, heightened infection can result in tree death [27,28]. Efficient seed-dispersing mechanisms and/or frugivorous avians, as well as highly diversified haustorial structures, contribute to their enhanced tropism [27]. These pests severely affect tree plantations across the globe [27,29]. In addition to being a keystone resource of biodiversity and, to a somewhat minimal extent, their medicinal assets, they have also raised serious concerns regarding the commercial fruit and timber farming communities [28,30,31,32]. In spite of the efforts of these and some research communities, conventional mistletoe management approaches have not succeeded in mitigating the mistletoe problem. On the contrary, most of these approaches, such as deliberate fires, herbicide use, pruning, pollarding, plastic wrapping, etc., overlook damages caused to the environment and public health [27,29,33]. To counter this, newer approaches followed, exploring hyperparasitism from biological entities that thrive on the mistletoes and testing their worth as mistletoe biocontrol agents (MBCAs). However, to date, no MBCA formulation has yet been translated to the market. Despite some silvicultural trials in small-scale settings, other biotechnological interventions were limited to time-consuming and laborious tree-breeding strategies [34]. Beyond these, transgenic approaches and smart solutions have not yet been explored in the 21st century. In the face of emerging new host records and enhanced tropism, mistletoe seems to be outperforming management initiatives and losing the necessary pace of development and advancement. 

This article examines the pros and cons of conventional mistletoe management and explores whether the biotechnological interventions have provided breathing room for biocontrol in terms of developing and using (i) biological mistletoe control agents and (ii) transgenic technologies to reduce the mistletoe burden on commercial fruit and timber tree plantations. The future possibilities with smart solutions for mistletoe management and the bottlenecks are also discussed. 

## 2. The Biology of Mistletoe

**Figure 1:**
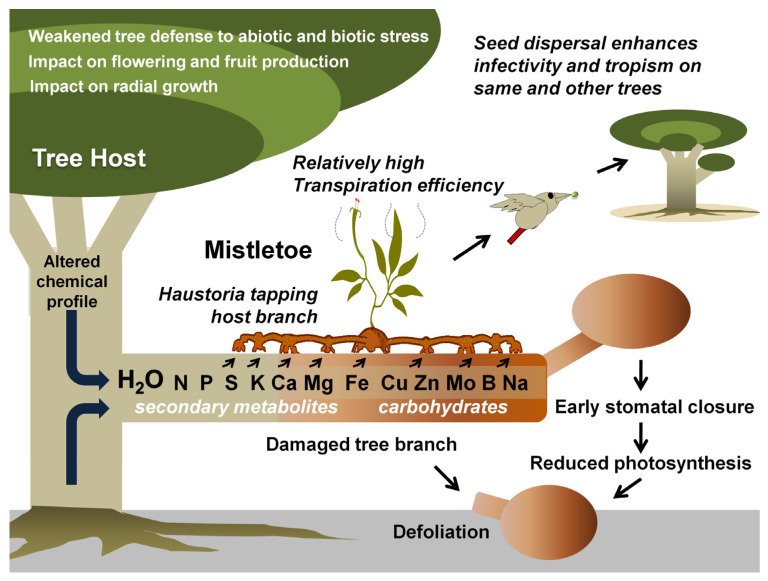
Mistletoe parasitism and impacts on a tree host. Specific mistletoe may parasitize its host by exploiting its branch(es) for water and various other resources, thereby limiting its photosynthetic potential, optimal growth, response to various stresses, and fruit or timber production. Gradually, infective spread (by haustorial connections and endophytic expansion) and growing infection instances (ensured by mistletoe seed dispersal) may lead to early senescence of the tree(s).

**Figure 2:**
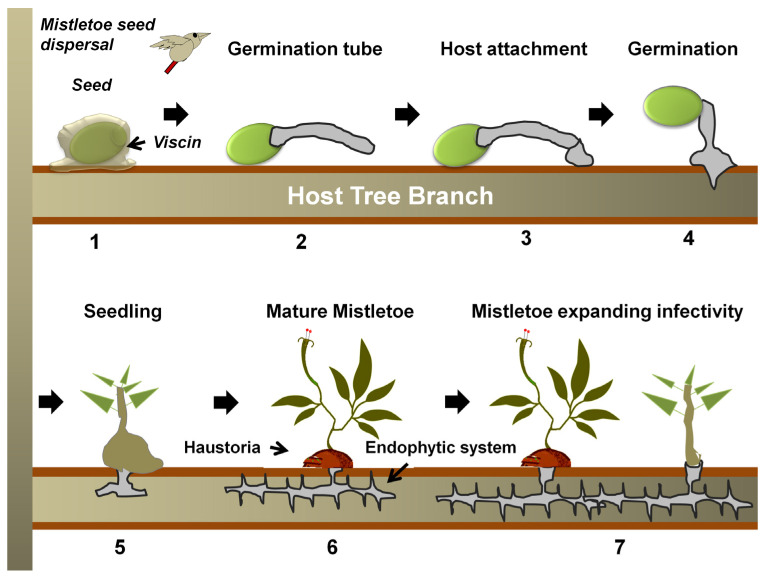
A generalized life cycle of a mistletoe parasite on a tree host. Following seed dispersal, a sticky material, viscin, over the seed coat ensures firm attachment to the host branch (in 1) [87,88]. Hypocotyl(s) from the seed then emerges and grows towards the bark (in 2), which eventually, while attaching to the bark (in 3), penetrates through it (in 4) [89]. Once the hypocotyl connects to the host xylem (in 5), it exploits the host for water, minerals, carbohydrates, and secondary metabolites (in 6) [83]. The endophytic system may expand as sinkers (or epicortical roots) along the host cambium, from which sporadically new shoots may emerge (in 7) and facilitate the heightening of infectivity [65].

Mistletoes occur in the order Santalales, occupying the Loranthaceae (approx. 1000 species) and Viscaceae (approx. 550 species) [35]. Notably, the most notoriously damaging species in the Loranthaceae are the honey-suckled (*Dendrophthoe* spp.), the showy (*Helixanthera* spp. and *Psittacanthus* spp.)*,* and the red mistletoes (*Tapinanthus* spp.), while, among the Viscaceae are the Dwarf (*Arceuthobium* spp.), the American (*Phoradendron* spp.), and the European mistletoes (*Viscum* spp.) [36,37,38,39,40,41,42,43,44,45,46,47,48,49,50,51,52,53,54,55,56,57,58,59,60,61,62,63]. Mistletoes are characterized as hemiparasitic plants because of their reduced photosynthetic efficiency and the absence of a true rooting feature [18,64]. A false root-like appendage, known as a haustorium, attaches them to their host plants (mostly trees) and draws water and nutrients from them [65,66,67] (Figure 1). Generally, these haustorial connections lack a retranslocation system, meaning that the hemiparasites directly and exclusively associate with the host xylem, but exploitation of the host phloem is never reported [68,69,70]. By transpiring almost nine times as quickly as their hosts, mistletoes suppress their host’s ability to maintain water potential, thus causing early stomatal closure and reduced carbon assimilation [11,64,71,72,73,74]. Hosts find it difficult to maintain their water, carbohydrate, and mineral profiles, especially under drought and soil infertility conditions [75,76,77,78]. In addition, climate change may add to the host detriments since mistletoe may spread to new geographical regions, possibly infecting new hosts and increasing in infectivity [71,75,76]. Mistletoe seeds are dispersed predominantly by fruit-eating birds [79,80]. Some birds have coevolved with mistletoes exhibiting fruiting displays [81]. Mistletoes attract a narrow range of avian dispersers that have anatomical adaptations and dietary preferences specific to mistletoe fruits [66,81,82]. Some *Viscaceae mistletoe* in the genera *Arceuthobium* and *Korthalsella* are equipped with explosive dispersal mechanisms in their seeds [66]. Seed dispersal by these modes has possibly allowed mistletoe to spread to nearby potential host trees, as well as those in islands and continents far off [83,84,85,86]. Seed dispersal on a compatible tree host marks the start of the mistletoe life cycle and parasitism (Figure 2). 

## 3. Mistletoe Damages Trees

There is a great deal of liability associated with mistletoe in recreational settings, as well as commercial losses [26,28,31,90]. However, only a few surveys have been conducted at global scales to obtain data depicting the extent of damages caused by individual family members [87,91]. Several billion dollars’ worth of timber is lost each year due to dwarf mistletoe in Western Canada and the United States [92,93]. Many species causing heavy losses were reported early in the 1970s [94]. European mistletoe (*Viscum album*) itself is known to affect around 450 varieties of trees [95]. In North America and Canada, dwarf mistletoes (*Arceuthobium* spp.) predominantly affect the coniferous forests, especially tree species such as western hemlock (*Tsuga heterophylla*), lodgepole pine (*Pinus contorta*), and Douglas fir (*Pseudotsuga menziesii*) [51,96,97]. In Australia, eucalyptus and acacia plantations are heavily attacked by *Amyema* and *Korthalsella* species [98,99]. In Asia, the Korean mistletoe (*Viscum coloratum*), *Macrosolen* spp., and the Indian honey-suckled mistletoe (*Dendrophthoe falcata*) are more prevalent [100,101,102,103].

Depending on the parasite’s size, proliferation rate, and photosynthetic potential, as well as the host’s resourcefulness and developmental phase, mistletoe parasites have varying impacts on their hosts [44,46,59,60,61,104,105]. From the first infection, whether mistletoes kill the host or whether other factors offset this mortality is debated [28]. Many studies suggest that mistletoes negatively impact tree survival and fecundity, resulting in changes in population dynamics and structure, which in turn threaten their sustenance and conservation status [98,106,107,108,109]. An infected host (Figure 1) may indicate water stress and depletion of macro- (N, K, Ca, P, and S) [64,110,111,112] and micro-nutrients (Mg, Fe, Cu, Zn, Mo, B, and Na) [113], growth retardation [61,114], morphological and physical abnormalities [62,63,115,116], and shortening of stomatal opening [71]. Upon intensified infection, these effects slowly but eventually lead to the premature death of the host [39,60,117,118]. Besides the above direct damages, changes in the metabolic turnover and water stress (imposed by a competitively higher mistletoe transpiration rate, especially in water-limited scenarios) may render increased tree host susceptibility to microbial pathogens [106,119,120]. Infection with these hemiparasitic plants results in the loss of approximately 22–43% of carbohydrates in hosts [121]. *Phoradendron perottetti* affects *Tapirira guanensis*, resulting in the loss of many primary metabolites (especially soluble carbohydrates) and secondary metabolites (high levels of flavonoids) at the galls [53,61]. This is indicative of reactive oxygen species (ROS) quenching [53,61]. Further, a reduction in chlorophyll, foliage, and the biomass of hosts may reduce the photosynthetic potential as well as host biomass [52,58,59,62,122,123,124,125]. The radial growth of tree hosts has been reduced by *Viscum* spp. in Germany, Switzerland, and Spain [111,123,126], as evident from the reduction in annual growth rings in trees [125,127]. Moreover, mistletoe can interfere with flowering and fruiting and make the hosts more vulnerable to insect and fungal damage [128,129]. They also compete with host chemical profiles to remain healthy [130,131]. Lázaro-González and coworkers showed that *Viscum album* may load-dependently reduce the nitrogen concentrations in its pine hosts and may effectuate defense signals resembling drought, burning, and defoliation [130]. Recent work with *Phoradendron crassifolium* and *Psittacanthus robustus* revealed that tree branches neighboring the mistletoe infection site relatively experienced more adversities [132]. Studies have also reported a 40% reduction in mycorrhizal richness in tree hosts during mistletoe infestation [133,134]. In terms of foliar appearance, mistletoes often mimic their hosts [135,136,137]. This prevents them from being recognized by predators and herbivores that feed on them [135,136,137,138,139]. The exploitation of secondary metabolites from the host is known to confer such properties [135,136,137,140]. By doing so, mistletoe bypasses natural removal from hosts and escapes pruning or pollarding practices. The mimicry hypothesis in the case of Australian mistletoes, however, was questioned in a study by Blick and coworkers [141]. 

All the above detriments may result in the premature senescence of commercially valued tree varieties, such as fruits, timber, and others. Control of mistletoe is limited because of many contributing factors, including easy seed dispersal by frugivorous birds, explosive seed dispersal mechanisms, and reinfestation with remnants post-pruning, as well as others such as costs, labor, seasons, and climatic factors. 

## 4. Conventional Control Strategies and Integrated Pest Management Approaches

Numerous conventional mistletoe control options have been routinely practiced by primary producers to mitigate mistletoe infestation. These include physical, chemical, and silvicultural approaches. We discuss the merits and downsides of these options below. 

### 4.1. Physical Methods

Mistletoe was managed by people in forests and farms using various primitive methods. In the US, western dwarf mistletoe (*Arceuthobium campylopodum*) predominantly affects Ponderosa and Jeffery pines in forests [90,142]. These mistletoes can reduce life expectancy and result in the poor growth of infected hosts, as well as a loss of their scenic and esthetic value [90,142]. Conventional practices such as pruning and thinning of the affected host parts have proven less helpful [28,143,144,145]. There are many concerns and problematic issues that also have hindered these methods [90,145]. Known practices that have shaped the integrated management regimens for the control of mistletoes have been categorized into direct and indirect approaches [29,90]. Direct methods include the individual removal of infected trees, branches, or mistletoe brooms, or installing buffer strips, and the use of hosts that can resist mistletoe parasitism [90]. These methods could limit mistletoe spread and infection rates to some extent [90]. The potential benefit of removing the infected branches in Scot pines (*Pinus sylvestris*) aids in evading the inherent competition for water and nutrients within the host plant, which concomitantly results in improved tree health, such as elevated height, biomass growth, and diameter [111]. The downside of the direct methods, however, has been the labor and cost-intensiveness, limiting their application to only local small and high-value areas including parks, city limits, orchards, and nurseries [146,147]. It may be possible to remove large areas of mistletoe-infested trees; however, scheduling this around the growth periods of various hosts would be difficult and time-consuming [148,149]. Additionally, there are many modes of seed dispersal, including birds, winds, and storms, which make these strategies difficult. For example, it is difficult to expect a dwarf mistletoe-free stand within approximately a hundred feet of an infected Ponderosa pine [90,150,151,152]. Reid and Yan [153] highlighted the possibilities of developing aerial, ultra-light, surgical, and/or chemical delivery options. In either case, such approaches may not efficiently eliminate mistletoes from heavily parasitized trees [153]. Surgical methods require time, labor, and financial costs, making them impracticable for small- to large-scale plantations [29]. Owing to expenses, farmers inevitably cannot employ professional services from arborists and tree surgeons who offer tree lopping and pruning options [29]. For them, the only option is to resort to the age-old traditions of pollarding, as in Southeast Australia [29]. This, in addition to mistletoe control, gives them fodder during droughts, shade for livestock, firewood, and fencing [29]. Other physical methods, such as the use of paints and deliberate fires, are viable measures [154]. Fire applications have offered some appreciable control of the mistletoe population in the western USA forests [155,156,157], because of mistletoes’ slow recovery rate compared to host(s) [158,159,160]. However, the use of fire may have limitations in that a weakened host may favor mistletoe regrowth [51,161,162]. Moreover, on holistic grounds, fire applications may raise public and environmental health concerns [163,164]. Indirect methods use irrigation, fertilization, brush and weed control, and regulating human disturbances [29,90,165]. Irrigation profiles of the host may variously impact hemiparasite infectivity. In many cases, the higher water status of the host corroborates with heightened mistletoe infectivity, hinting that intermittent drought regimes would offer some protection for the hosts [90,166,167,168]. Improving the fertility of hosts by the use of nitrogen fertilizers has shown some protection from the mistletoes’ effects, with increments in tree height [169]. Other indirect approaches also aid in improving host vigor and thus can be applied in large settings [90]. Above all, scientific evaluation of the above strategies requires surplus funds and support for research and development communities to devise containment facilities and flexibilities in regulations to work in protected forest areas [29]. 

### 4.2. Chemical Methods

Though with only limited prospects, chemical-agent-based approaches to mistletoe control have been more prevalent in various non-European countries such as India, Bangladesh [57,170], Australia [171], and the United States [88]. These methods involve the use of sprays or trunk injections of certain herbicide formulations to selectively control the hemiparasite without negatively impacting the host plant species [171,172,173,174]. In Eastern Australian farm eucalypts, the use of 2,4-D formulations had been investigated long ago in the 1950s and 1960s to control box mistletoes (*Ameyema* spp.) [174]. Besides their poor control, the trials could not be widely extended or reproduced with equivalent success due to the difficulties in ascertaining the optimum dose as influenced by various factors such as mistletoe host species, its size, the season of injection, the frequency of mistletoe infestation, and others [171,172,173]. Formulations such as 2,4-D, 2,4,5-T, 2,4-MCPB, and dichloroethane have substantially been utilized to destroy *Viscum album* subsp. *abietis* on Abies with only insignificant host effects [34]. However, complete resistance to mistletoe is never induced in hosts, as seen in the case of using 2,4-D and glyphosate, wherein mistletoe control only lasts half a year post-treatment [34,175]. A more promising herbicide control measure, according to a large study by Quick [176], involves using an iso-octyl ester of 2,4,5-T, which was later banned for its non-target effects [34]. It was shown that numerous other formulations tested between 1970 and the mid-1990s, such as Nutyrac, Dacamine, Thistrol, MCPA, Goal, D-40, Emulsamine, DPX, Prime, and Weedone, were unreactive to the endophytic system, despite killing mistletoe foliage exclusively, without adverse effects on the host [128]. Similarly, ethephon (Florel, 2-Chloroethyl phosphoric acid), which releases ethylene, a plant growth regulator, has been tested with various dwarf mistletoe–host models in Manitoba, California, Idaho, New Mexico, Oregon, and Minnesota [177,178,179]. With few non-target effects, it results in defoliation in mistletoe, albeit without influencing the haustorial connection, meaning that the complete evasion of infection is still a concern and recurrence cannot be ruled out [180]. A defoliated but active haustorium can persist for over a century [87].

It is still necessary to ensure the prompt delivery and adequate coverage of chemical agents, even if they appear potent enough [26,34,181]. Ground applications can be effective but may not reach the hemiparasites in the tree overstory higher up in the canopies [34]. Helicopter-based aerial delivery approaches, however, are challenged by poor penetration to the lower crowns, as sprays would be hindered by the overstory crowns [182,183]. To continue, chemical control methods pose many difficulties, such as limited technologies for the selective treatment of mistletoe over hosts, chemical damage to hosts, unavoidable reinfestation due to parasite seed germination or spread after chemical application, developing crops with a high tolerance to chemical applications, low penetration, persistence, availability, and so on [180,184]. Again, chemical agents that do not harm the haustorial connections may gradually exhibit resprouting [185]. This will be graver in areas with greater incidences of mistletoe infection and its resprouting-led intensification [185]. Despite the best chemical agents, mistletoe seeds will only be delayed for 2–4 years, so there is only a temporary solution to the problem [34]. Considering these factors, alternative control and management methods are highly sought after. 

### 4.3. Silvicultural Practices

In contrast to the above approaches, silvicultural practices have been more promising [90,128,148]. These work by first clearing mistletoes from infection sites and then reintroducing a fresh lot of plants to the area [186]. Such methods have been implemented for controlling dwarf mistletoes in North America [128,186]. Robinson and colleagues proposed a spatial–statistical model that simulates mistletoe growth and development under many control methods and offers a more preferable option [187]. 

In small recreational and commercial settings, a silvicultural approach focuses on multiple factors, including ecological disturbances, harvests, prescribed fires, and need analysis and impact assessments [148,186]. They have gained acceptance as a routine strategy for highly devastating mistletoe and have slowed the development of resistant cultivars [34]. Silvicultural practices, however, may not guarantee long-term and consistent efficacies due to the gradual changes in climate and shifts in devastating mistletoes related to their enhanced tropism [186]. In order to scale this approach and ensure its success, newer options are highly desirable. A recent investigation showed an associational resistance to the spread of *Viscum album* ssp. *austriacum* in Northern Spain and indicated that mixed plantations of hosts (Scot pine, *Pinus sylvestris*) with non-host (Maritime pine, *P. pinaster*) trees greatly reduce mistletoe infestation [188]. 

## 5. Mistletoe Control through Biotechnological Interventions and Smart Management

Biotechnological approaches and the use of 21st-century technological advancements can bring a paradigm shift to the current mistletoe management regimens. Mistletoe control biotechnology should realize the use of potential MBCAs, which variously deteriorate and kill the mistletoes on tree hosts (Supplementary Table S1). Alternatively, enhancing or introducing traits conferring mistletoe resistance in host trees should be considered a more promising option [189]. The MBCA approach has seen limited application in finding and characterizing potential microbial and insect pathogens, as well as other life forms, but is yet to see a fully mechanized industry setup and global application [190]. The genetic approach similarly relies on mere conventional breeding trials [128,191]. In the sections ahead, we examine the current status of these highly promising biocontrol options and develop various plans to take advantage of them. 

### 5.1. Mistletoe Biocontrol Agents (MBCAs)

Organisms with specific nutritional requirements that jeopardize their concomitant host(s), have emerged as a biocontrol strategy in most pest management routines [24,192,193]. Integrated pest management practices have become an established part of agriculture, forestry, and horticulture [194]. Such biocontrol agents offer wide acceptability and ease of application due to their straightforward and easy production processes and various biosafety and eco-friendly attributes [195]. In particular, for use as MBCAs against dwarf mistletoes, various fungi, bacteria, and insects have been proposed [196,197,198,199,200]. 

#### 5.1.1. Bacteria and Fungi as MBCAs

Many well-known marketed mycoherbicidal formulations are available as MBCAs for controlling woody weeds, viz., DeVine^®^ (*Phytophthora palmivora* for controlling strangler vine on citrus) [201], Collego^®^ (*Colletorichum gloeosporioides* for controlling Northern Jointvetch in rice and soybean crops) [202], and BioMal^®^ (*Colletotrichum gloeosporioides* for controlling round leafed mallow weed in agricultural settings) [203]. *Chondrostereum* *purpureum*, a primary wood invader, has been investigated by Canadian and European researchers as a biocontrol solution for undesirable woody weeds in conifer regeneration and forestry settings [204,205,206,207]. Similarly, StumpOut^®^, a registered biocontrol product, is a white-rot fungus (*Cylindrobasidium laeve*) used in South Africa for controlling Australian wattles [208]. As with weeds, hemiparasitic mistletoes also harbor a range of microbial pathogens with varying species specificity. Hyperparasitism from both fungi and bacteria (hence myco- and bacterio-herbicides, respectively) can be used for mistletoe biocontrol, some of which are listed in Table S1 (Supplementary Material). Fungal pathogens reported on many Australian Loranthaceae mistletoes [209] receive proper attention regarding their potential as MBCAs. To explore control over dwarf mistletoes, the myco-MBCA candidates are considered in two categories: the aerial shoot fungi that act on the foliage, and the canker fungi, which attack the endophytic system [210]. More than 20 different canker types are known for *Arceuthobium tsugense* in British Columbia; however, because their MBCA potential includes both pros and cons, they require in-depth assessments before mass field application [87]. Nevertheless, three potent canker fungi are described. American and Canadian dwarf mistletoes (*Arceuthobium* spp.) commonly harbor a fungus, *Colletotrichum gloeosporioides*, which has been investigated for its potential as a mycoherbicide MBCA for controlling this mistletoe on western hemlock and lodgepole pine [210]. This fungus is virulent, easily cultured in vitro, and completely disrupts mistletoe growth shortly after shoot emergence, and it has shown success in field trials attempted in British Columbia. Especially for *A. americanum* and *A. tsugense*, in vitro assays have been developed to evaluate the potential of this fungus as an MBCA [211]. Other than this, a canker fungus, *Neonectria neomacrospora*, has also been isolated with documented pathogenicity from cankers caused by *A. tsugense* in British Columbia [212]. In addition to its ability to infect its mistletoe host without wounding, quick sporulation, selective spread, foliage-reducing properties, and mortality characteristics, the fungus is selective to infect dwarf-mistletoe-infected host tissues. At field sites for dwarf mistletoe, intensive efforts are being made to optimize and improve industrial-scale production and delivery methods for this MBCA [213]. The anamorph-staged fungi Cylindrocarpon has been tested in vitro as another, yet more promising MBCA (than *C. gloeosporioides*), with aggressive colonization on dwarf mistletoe seedlings and callus [211]. *Cytospora abietis* Sacc. is a well-known fungus attacking dwarf mistletoe cankers on red and white fir species *Abies magnifica* and *A. concolor*, respectively [214,215].

*Arceuthobium americanum*, parasitizing *Pinus contorta* of the Rocky Mountains in Northwestern America, commonly succumb to Resin Disease Syndrome, a disease outcome of a complex attack from various fungi, which predominantly includes *Alternaria alternata*, followed by *Aureobasidium pullulans* and *Epicoccum nigrum* [216]. This can result in excessive resinosis of the haustoria, necrotic lesions in the bark, and dying foliage as symptoms potentiating MBCA candidature for each of the pathogens. However, these fungi have also been isolated individually from non-symptomatic mistletoe–host associations, indicating that the syndrome is a multipathogenic phenomenon [48]. Substantiating an MBCA, potentially of individual fungi or one MBCA cocktail formulation, hitherto demands further investigations on reproductive potential, the comparison of systematic and non-systematic infection in mistletoe, and the characterization of environmental and fungal components as well [216]. 

*Viscum album* negatively affects a variety of pine trees in Turkish forests, which are important timber crop species [105]. Kotan and coworkers [217] analyzed the MBCA efficacies of several bacterial and fungal isolates from the foliage of various diseased *V. album*. They reported five possible bacterial MBCAs [217], which were pathogenic only when administered to mistletoe through injection, and not when sprayed. In addition, they reported four highly pathogenic fungal strains that showed potential for MBCA when sprayed. Very recently, *Aureobasidium harposporum*, a new fungus, has been isolated, which causes leaf spot disease on *V. album* in Turkey [218]. 

#### 5.1.2. Diatoms and Algae as MBCAs

According to reports, diatoms and marine algae resist parasitic bacteria, fungi, and insects [219,220,221,222,223,224]. However, very little is known about their efficacy in controlling parasitic plants. In a recent study, a commercial diatomaceous earth formulation, Mistletoe Killer^®^, was reported to parallel ethephon in controlling the mistletoe *Arceuthobium globosum* ssp. *grandicaule* parasitizing *Pinus pseudostrobus* [47]. Accordingly, a 7.5% dose of this formulation resulted in foliar death and complete mistletoe removal (respectively, after 15 and 45 days of application), without impacting the tree host. However, follow-up studies aimed at understanding the basis of the selective damage to mistletoe from diatoms remain to be pursued. It also remains to further test this formulation on other mistletoe–host pairs, which could validate the MBCA potential of diatoms and other algae’s application. 

#### 5.1.3. Insects as MBCAs

Several insect classes have the potential to become successful MBCAs. In 1984, J.D. Solomon, an entomologist, reported a stem borer weevil, *Myrmex* sp. (Coleoptera: Curculionidae), from the declining clusters of the mistletoe *Phoradendron serotinum* occurring on many water oak trees (*Quercus nigra*), near Stoneville, Mississippi [225]. This weevil tunnels into the mistletoe stem and deposits its larvae, which pupate and emerge, making holes and galleries within the stem, leading to mistletoe diebacks upon heavy weevil attacks. Before this, other species in this insect genera, *M. arizonicus*, and *M. algerti*, were also reported from mistletoes in the United States [226,227]. Recently, another phytophagous weevil, *Timorus sarcophagoides* (Coleoptera: Curculionidae) [228], has been reported to feed and carry out its life cycle exclusively on the parrot flower mistletoe, *Psittacanthus robustus* (Loranthaceae). It feeds on soft tissues in the flower buds, pollen grains, stamens, ovaries, and leaf axils, as well as digging tiny holes in the haustoria to oviposit and lay eggs. Adults are known to walk throughout the mistletoe and rarely leave it. Moreover, by closely resembling flesh flies (Diptera: Sarcophagidae), these adults represent a valuable example of evasive mimicry, enabling them to be overlooked as uncatchable prey by birds and other predators [229]. For use in MBCA, this property may ensure extended consistency of application.

*Loranthus* spp. predominantly attack tea, citrus, guava, rubber, kapok, rubber, and eucalyptus plantations in many parts of the world [87]. In two old monographs documenting a cursory survey of phytophagous flora in West Pakistan, Mushtaq and Baloch have reported many species of insects and mites infesting *Loranthus longiflorus* [230,231]. These researchers also explored the mistletoe specificity in insects to account for their suitability as an MBCA. Of these insects, some were never reported for any alternate host, while others were more destructive to the tested mistletoes, proving their worth as putative MBCAs. These were oligophagous in their restricted inhabitance to Loranthaceae mistletoes.

Some insects complete part of their life cycle on mistletoes in the form of egg deposits [225,226,227,228]. One such family is Buprestidae (Coleoptera) of the Jewel beetles [232,233]. They include *Agrilus viscivorus*, *A. graceus*, *A. jacetanus*, and *A. kutahyanus* [232,233], all of which thrive on mistletoe sap and show a detrimental influence over the mistletoes, eventually killing them upon their maturation. 

Moths, such as *Synanthedon loranthi* (Lepidoptera: Sesiidae), have also been found harboring mistletoes that infect *Scots pine* [234]. The larva burrows into the mistletoe stem and pupates on the leaves [234]. Some moths belonging to the family Tortricidae, such as *Celypha woodiana* and *Ditula angustiorana*, feed over the mistletoe leaves [235,236,237]. Hemipteran moths such as *Cacopsylla viscid*, *Anthocoris viscid*, and *Pinalitus viscicola* have also been reported on mistletoes [238,239]. 

In another case, scale insects have been evidenced to cause disease symptoms over mistletoes. False oleander scale insect (*Pseudaulacaspis cockerelli*; Hemiptera: Diaspididae) thrives over the honey-suckled mistletoe (*Dendrophthoe falcata* var. *falcata*; Loranthaceae) found parasitizing the leguminous Cassia trees (*Senna siamea*) in India [37]. Other than this, a cottony cushiony scale insect (*Icerya purchase*; Hemiptera: Monophlebidae) was reported exclusively to be infective on the red-berried mistletoe (*Viscum cruciatum*), and not the mistletoe host, the olive tree [240]. Importantly, tree hosts in both cases were uninfluenced by scale insects, meaning that the latter could be selective to mistletoes. 

There is a need to carry out faunistic surveys and sampling on various mistletoe–arthropod associations so that enough resources are available for lab-to-field-scale tests before reaching a conclusive remark on the candidate MBCAs of the insect world [241]. Similarly, many of the previously reported insect fauna from the New World dwarf mistletoes in Asia require detailed studies [242]. 

#### 5.1.4. Hyperparasitic Mistletoes

Some mistletoe species may parasitize other mistletoe species growing over tree hosts. This is termed hyperparasitism [243]. For example, the Bollean mistletoe (*Phoradendron bolleanum*) usually grows on trees (in the genera *Juniperus* and *Arbutus*), but occasionally appears as a facultative hyperparasite on various mistletoe species [244]. *Viscum capitellatum* in Sri Lanka is an obligate hyperparasite of widespread mistletoe *Dendrophthoe falcata* L.f. [245]. There are many examples of hyperparasitism in both Loranthaceae and Viscaceae [55,246,247,248]. Plant phenology and seed dispersal agents’ behaviors play important roles in the chance occurrence of hyperparasitism [243]. However, developing such MBCAs would be illogical and difficult in managing (i) the technical diligence of their delivery for controlled release and (ii) off-target effects and concomitant outbreaks. In addition, it may switch to parasitizing the host tree, which would otherwise be rescued and restored from mistletoe attacks. The realization of such an approach would also require a deeper understanding of mistletoe’s compatibility with hosts, epigenetic influences, and other factors.

#### 5.1.5. Higher Animals

Higher-order organisms in the animal kingdom may aid in devising mistletoe control measures [29]. Common bushtail possum (*Trichosurus vulpecula*) is a leaf-loving marsupial native to Australia [249]. In a conference proceeding by Sessions and co-workers, this mammalian species was reported accountable for the decline of the endemic Loranthaceae mistletoes *Peraxilla tetrapetala*, *P. colensoi*, and *Alepis flavida* [250]. Around 50% of the studied *Peraxilla* mistletoes were defoliated by the possums between 1978 and 1982 at Australia’s Nelson Lakes National Park [250]. This could pave the way to a newer strategy to use such animal species in countries where mistletoes heavily damage commercial fruit farms and forests [251,252]. However, this control option could be challenged by the declining habitats for these animals onsite [25]. 

### 5.2. Requirements of an Effective MBCA Selection Program

**Figure 3:**
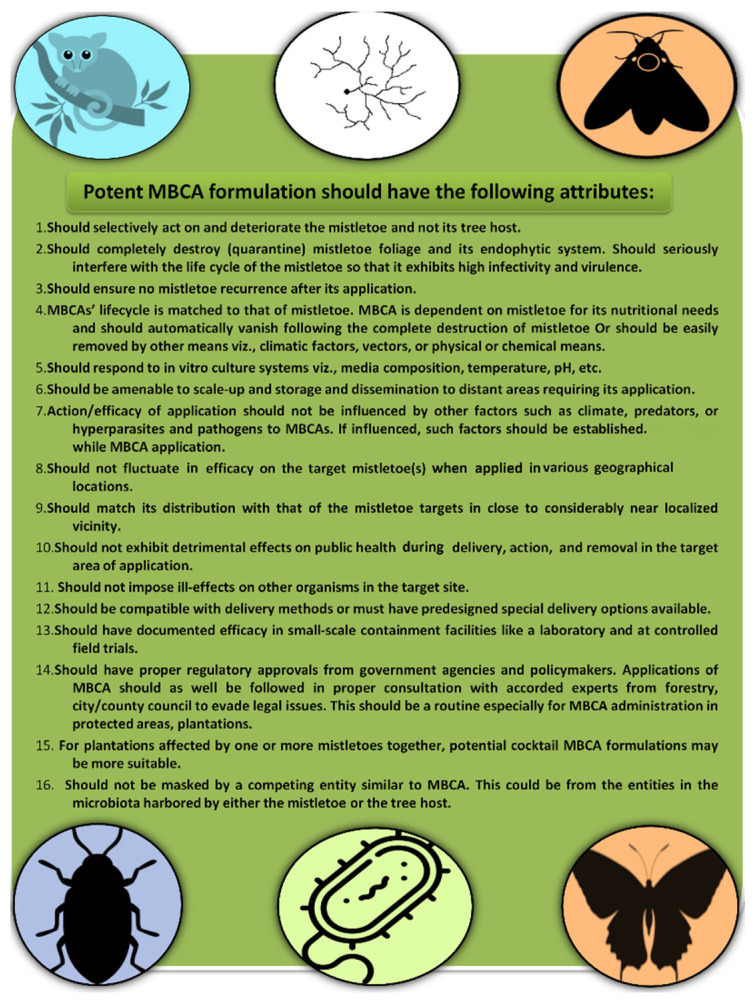
Selective traits for testing and screening of potential MBCAs. The infographic depicts a generalized checklist of features desirable for testing and screening of putative MBCAs. A more detailed checklist should include features sought for MBCA formulations of specific organismic entities, viz., microbes, insects, etc. Some features in the above are adapted from known monographs [216,255].

A comprehensive investigation of MBCAs may require expansive sampling and surveys towards generating metadata on biological entities, viz., insects and microbial communities that selectively impose pathogenic symptoms on mistletoes without harming their respective tree hosts. Future research should focus on individual entities and on characterizing the performance of emerging MBCAs against specific mistletoes. There is a need for rigorous research on the synergistic effects of two or more potential species (which could create an MBCA cocktail) for more notorious mistletoe(s), areas complexed with more than one variety, or for those that attack multiple hosts at the same time. Examples of primitive studies of this type exist [253,254]. Insects have equal potential as microbes for use as MBCAs, as many in their larval form feed exclusively on mistletoes [230]. In terms of MBCA potential, mistletoe pathogenicity alone cannot provide a concrete solution. Before selecting MBCAs, a checklist of features that best suit the testing of putative entities should be developed. An ideal checklist should include all the possible critical attributes on which MBCA hyperparasitism can be tested on acid, with a scoring system for their efficacy inherently (as MBCA) and their impact on the mistletoe, its tree host, and other non-host plantations. MBCAs would also require mass production, effective delivery systems, deployment strategies [34], and a consistent monitoring program. Below is a comprehensive checklist of parameters that can be used to test and screen MBCA candidates (Figure 3). 

### 5.3. Challenges with MBCAs

A different perspective considers pathogenic microbes and insects as co-evolving complex relationships with mistletoe hosts [34]. Mistletoe outbreaks are induced or regulated by a variety of factors associated with seasonal and weather variations and their influences on multitrophic communities [34]. This emphasizes the importance of intensive surveying, isolation, and study of the mistletoe–host specificity for each of the biological entities carrying considerable potential as an MBCA. Perhaps many microbes may appear circumstantially sensitive even to minute changes in their growth parameters [256,257]. The culturability and preservation (and conservation) of such organisms is also very important for reproducibility in continued/extended studies and applications. Many of these might be new, yet unexplored, rare, or others nearing extinction regarding strains, varieties, species, and of course their MBCA attributes. Moreover, some of these putative MBCAs may be lost due to environmental calamities and/or seasonal variations, especially for those very specific to the endemic mistletoes (especially their varieties). This must hold for entities that have no alternate host(s) than mistletoes. Examples of this could be the fruitfly genus, *Ceratitella*, represented by only four species, all of which thrive exclusively on mistletoes [258], or moths such as *Zelleria loranthivora *(Supplementary Table S1). Potential MBCAs are rare due to the scarcity of hosts that would otherwise enable their ecological expansion on more than one or very few mistletoe species. This seems imperative, especially for those endemic to not-so-vast geographic areas, where MBCA entities might lose some of their importance and worthiness if physical and chemical treatments are practiced in these areas. This will be more serious for entities that are closely related to, dependent on, or paralleled with mistletoe hosts. Mushtaque and Baloch’s study, for example, could not test six of the twelve insect species for MBCA potential [231]. The conservation and production of rare isolates may represent new technical challenges, such as strain- or species-specific media compositions, microbial culturing, and insect-rearing protocols. In addition, selecting MBCAs would pose the challenge of competing with other members of the host–mistletoe microbiome, as has been highlighted recently [259]. 

### 5.4. Inducing Host Plant Resistance to Mistletoes and/or Herbicides

Inherent resistance to mistletoes in some trees, such as Chinese pistachio (*Pistacia chinensis*), crape myrtle (*Lagerstroemia*), sycamore (*Platanus occidentalis*), and conifers such as cedars (*Cedrus*) and redwood (*Sequoioideae*), has been documented amongst their susceptible genotypes [260]. A call to develop a controlled breeding program catering to the identification of genetic bases for inherent resistance in tree hosts to hemiparasitic mistletoes was made as early as the 1960s [189]. Due to the advent of cost-effective and convenient silviculture solutions, it has received little attention from the scientific community [128]. Understanding the interactions of host–mistletoe compatibility would allow the selection of resistant varieties. Conventional breeding strategies, nevertheless, have developed some stably resistant tree lines [261]. Tree hosts may be evaluated for resistance to herbicides, which may aid in the better application of chemical control approaches following herbicide translocation through such trees to their mistletoe parasite(s). For many tree crops, breeding programs are time-consuming, labor-intensive, require extensive field trials, and have not resulted in herbicide-resistant cultivars [262]. Moreover, the chemical treatment of mistletoes on herbicide-resistant tree hosts might lead to the emergence of herbicide-tolerant mistletoes. 

### 5.5. Hunting for the Genetic Basis to Hosts’ Inherent Resistance to Mistletoes: Background Studies

Because mistletoes have been co-evolving with their tree hosts for nearly 25 million years [263], the emergence of inherent resistance in these hosts cannot be denied [264], especially for the very native and devastating mistletoes [265,266]. A genetically directed effect is also implied by the fact that mistletoes exhibit specificity to their concomitant hosts, as well as variability in host preferences [36,64,165,247,267,268,269,270,271,272,273,274,275,276]. For example, *Arceuthobium douglasii* does not parasitize *Pinus ponderosa* [128]; around 70% of dwarf mistletoe species with a principal host also pathogenize other hosts and still with variable levels of symptoms [191]; and *A. pusillum* fluctuates in terms of infection extent when exposed to *Larix laricina*, *Picea glauca, P. rubens,* and *Pinus strobes* [277]. On the contrary, there are also reported instances where mistletoes, in a heavily infected area, skipped infecting their principal host. A few observations were reviewed previously for *Arceuthobium* spp. [34] and one for *Dendrophthoe falcata* more recently [36]. However, studies are yet to present convincing demarcating features of preferred and not-so-preferred host type(s). These reports suggest existing variations in exhibiting resistance within the host population, even though the progeny of these trees have not been tested for resistance (a hotspot for further investigation). It is evident from these findings that few data are available regarding species-specific susceptibilities, which calls for rigorous field examinations such as progeny testing. Grafting techniques with putative resistant tree hosts used the inoculation of grafts and/or out-planting in mistletoe-infected sites [265,278]. Based on these trials, it appears that resistance is genetic rather than environmental. However, the heritability of this genetic regulation could not be documented. Many progeny tests to elucidate the heritability initially met with mixed results, with some escape or non-inheritable events [128,279,280,281]. Positives incidences [265,266,282,283] and later isozyme data from Nowicki’s work (as reviewed in Shamoun and DeWald [34]) more strongly support heritability. 

### 5.6. Mistletoe Community Restructuring, Disturbances, and Biological Interactions

Mistletoe infestations drastically differ at various geographic locations, and as such from urban to forest settings [275,284,285,286,287]. Their prevalence in these areas can be variously influenced by the occurrence of host species [276], host specificity [275,285], the behavior of dispersing agents [79], pollinators [288], habitat disturbances [154,289], herbivory [138], and chemical interactions with the hosts [290,291]. Mistletoes may be differentially prevalent and infective from one to another host and within a population(s) of hosts [292,293]. Generalist mistletoes have a broad host range, while specialists exhibit host preferences [64,109,275,294]. Moreover, community dynamics profoundly impact parasite–host relationships [79,80,108,269,270,295]. Numerous life forms interact directly or indirectly with mistletoe, exhibiting either generalist or specialist behavior in doing so [40,79,80,81,83,84,296,297,298]. Thus, field surveyors must consider influences under various circumstances before taking into account mistletoes’ infectivity, host preferences, and inherent resistance in the hosts within a particular geographical area (especially dense forest settings and commercial tree plantations). Vegetation disturbances such as the incidences of dead trees, grazing, logging, forest fires, and human waste disposal, among many others, may both positively and negatively affect mistletoe prevalence [50,299,300,301,302]. Other than these, mistletoes may differ in their ability to readily establish seedling growth and mature persistently on various hosts [273,303,304]. Mistletoes such as *Psittacathus calyculatus, Dendrophthoe falcata,* and *Phoradendron californicum* associate readily with leguminous tree hosts [37,39,305]. Mistletoes’ diversity and evolution may be effectuated by host shifts, followed by host specialization, as well as variations in climate and environment [306]. In addition to these factors, mistletoes’ success as parasites also depends on their complex interactions with herbivores, pollinators, and seed dispersers, such as birds, insects, and bats [40,41,298,307,308,309,310]. Many also believe that the genetic structuring and isolation of mistletoe into populations growing on various hosts within a community is attributed to these avian gene dispersal vectors [41,42,311]. However, based on the notion that most of these agents are insufficiently specialized, some believe that mistletoe speciation is mainly driven by host–parasite interplay [274,296,297,306,312,313,314]. Inconsistencies in mistletoe frequency within host populations are primarily due to seed dispersal mechanisms and disperser behavior. Some mistletoes (such as *Arceuthobium* spp.) exhibit explosive or ballistic seed dispersal [83]. Bird-dispersed mistletoes generally show a patchy distribution, which is attributable to birds’ eating, roosting, and nesting behaviors [39,54,315,316,317]. Forests with endemic marsupial seed dispersers may hold a similar analogy for effects on mistletoe prevalence and distributions [296,318,319,320]. Many host factors, such as tree diversity [86,273], height, crown width [43,49,295,321,322,323], and bark type [267], as well as the recently studied stand characteristics [105], regulate mistletoes’ colonization and density. These factors may also variously influence seed disperser behaviors. For example, the Sonoran desert mistletoe (*Phoradendron californicum*) seeds are reported to be less frequently deposited on host tree species *Cercidium microphyllum* and *Acacia constricta* [305]. The dense, thorny, and entangling crowns in these trees probably do not facilitate the perching behavior of avian dispersers [305]. Additionally, competition among the host trees may reduce resource availability and negatively impact mistletoe infection and occurrence [78]. Hosts may influence the reproductive phenology of their mistletoe parasites [324]. This could be indirectly orchestrated by host-mediated influences over the pollinator communities with distinct pollinator rewards and those over mistletoes with pollen receipts, as well as avian visitations from distinct taxa on distinct hosts [325,326].

Besides influencing the interactions above the ground, mistletoes also tend to restructure the communities in the soils underneath their hosts and act as facilitators of heterogeneity, especially in low-productivity areas [327,328]. They may contribute to a more nutrient-rich litter from the abscission of their leaves, fruits, flowers, and seeds, which decompose faster and enhance soil-nutrient cycling. The litter may pave the way for newer forms of community interaction. For example, Hódar’s group reported that *Viscum album* litter may favor herbaceous vegetation and frequent visitations by rabbits underneath their host trees [329]. Another study shows that mistletoe-remodeled soil litter can improve habitat quality for insectivorous birds that prefer specific arthropod populations underneath mistletoe host trees [330]. As a result, nearby tree hosts (mistletoe-preferred and non-preferred) become more competitive. Other than these, mistletoes can generate non-tropic and trait-mediated detrimental indirect interactions with insects sharing the same tree host [331]. The mistletoe can therefore affect the entire herbivore community and their dependents, such as insectivorous birds and other plants, some of which may serve as seed dispersers and pollinators. Alternatively, mistletoe infection may, directly and indirectly, influence defense systems in their hosts to natural enemies. This has been reported in some pine trees exhibiting resin duct defense against bark beetles [332]. Mistletoe-inflicted changes to host chemical profiles may even influence insect behaviors and drive their evolutionary transition to other hosts. This is exemplified in a study that reports the role of dwarf mistletoe (*Arceuthobium* spp.) in mediating butterflies’ (*Nephasia* spp.) interaction with their pine hosts [272]. Climate change, on the other hand, also may variously impact the global distributional shifts in mistletoe [166,167,333,334]. These factors likely impede field investigations that could otherwise identify genetic controls of mistletoe resistance. In this case, specially designed containment facilities such as glass or net houses may offer robustness to field studies. Moreover, research on heritable genetic factors is lacking in the literature. It is still unclear whether the resistance phenotype in the host is governed by specific genes and/or is a cumulative effect of genes encoding other traits. Some studies hint at the latter possibility of resistance resulting from anatomical changes in the response to aging and wounding [265,277,283]. Alternatively, exploring the possibility of genetically evolved secondary metabolites in resistant hosts for defense against mistletoes also might be promising [268]. These represent gaps in existing studies, especially regarding the functional genomics of mistletoe–tree host associations, which could translate into their genetic manipulation and engineering for stably resistant transgenic trees. We discuss these opportunities and others in the sections ahead. 

### 5.7. Transcriptomic/Metabolomic Profiling, Transgenic Trees, and Translational Research Pipeline

**Figure 4:**
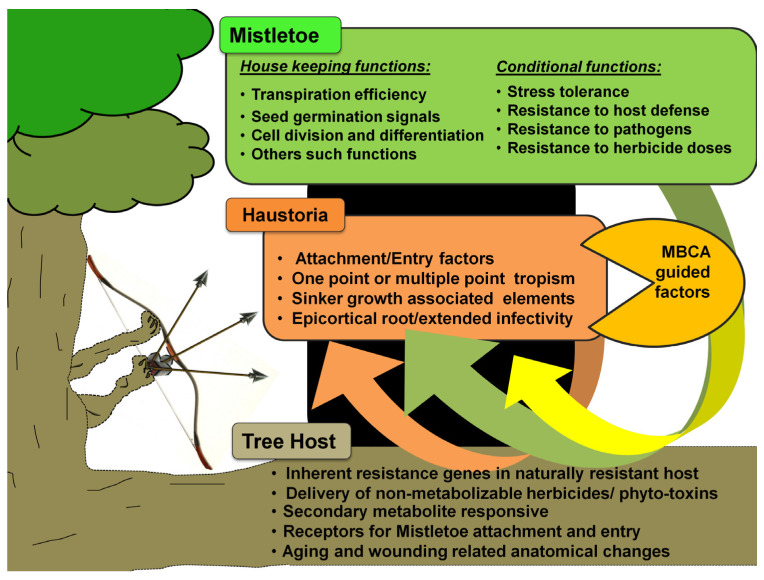
Resource-tapping possibilities from genetic pools to strategize transgenic host resistance to mistletoes. The gap in functional genomics studies on mistletoe parasitism has majorly limited the exogenous induction and/or introduction of mistletoe resistance in hosts. This would require, in principle, the characterization of genetic targets in mistletoe and tree hosts that signal and favor easy entry to mistletoes (shown here) and then testing of the conventional gene manipulation and transgenics’ frameworks (see Figure 5). Even putative MBCAs can be screened for genetic elements encoding factors detrimental to mistletoe parasitism and/or its life cycle.

The genetic modification of mistletoe hosts to induce resistance, resulting in a new variety, could be a promising strategy for reducing damage [335]. This would require the development of screening programs to characterize the viable sources of host resistance to each of the mistletoes (Figure 4). Such screens can be examined for molecular DNA tags, aiding in expedited marker-assisted selection for mistletoe-resistant hosts. Nonetheless, limited but considerable progress has been made in this direction [34,336,337,338,339]. Metabolomic profiling in other parasitic plants indicated the upregulation of auxin-responsive genes at the host–parasite interface [340,341]. Ichihashi’s group recently carried out both transcriptomic and metabolomic profiling of haustoria in the root hemiparasite *Thesium chinense,* revealing upregulated transcripts for genes involved in the biosynthesis and signaling of very long-chain fatty acids, auxin, and lateral rhizogenesis [342]. Along similar lines, Wang’s group attempted tissue-specific transcriptomics and whole-genome expression profiling of the shoot, flower, fruit, and seeds of spruce dwarf mistletoe (*Arceuthobium sichuanse*), revealing 22,641 differentially expressed genes related majorly to photosynthesis, carbohydrate and cell metabolism, transcription, hormone biosynthesis, and their signaling [343]. Recently, Wahid’s work extensively used tools such as suppression subtractive hybridization (SSH) and next-generation sequencing (NGS) to report some 1279 genes in Ziarat junipers (*Juniperus excelsa*) in Balochistan, which might putatively confer resistance to the dwarf mistletoe *Arceuthobium oxycedri* [344,345]. Transcriptome profiling of *Taxillus nigrans* individuals on four different tree hosts revealed many host-specific and common pathways driving this mistletoe’s parasitism [346]. 

**Figure 5:**
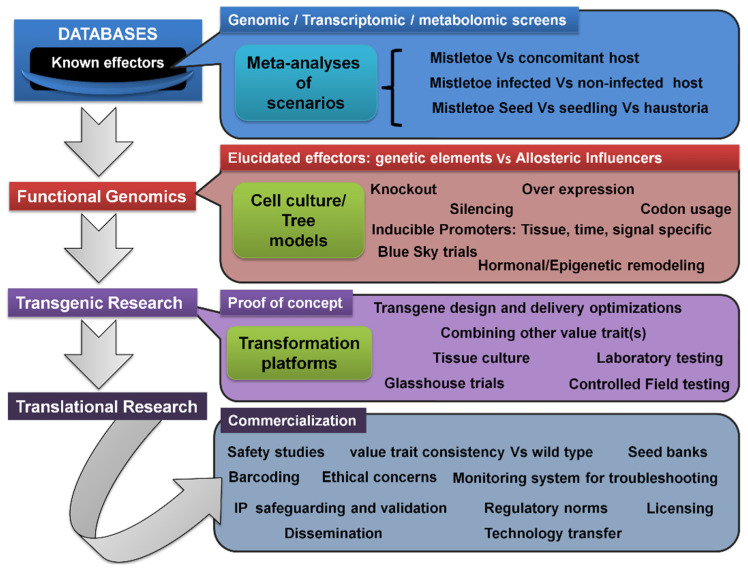
Generalized transgenic and translational research pipeline for developing and disseminating stable mistletoe-resistant tree hosts of commercial value(s). Genetic elements (or their homologs) putatively conferring mistletoe resistance may be recovered from relevant repositories such as the NCBI’s GenBank and KEGG, and also may be screened using systems biology and bioinformatics tools, following genomic, transcriptomic, and metabolomic analyses. Some suggestions are depicted in Figure 4. Precisely, such meta-analyses can be performed on various pairs of genetic/expression pools. Once the genetic elements directly encoding the resistance trait or indirectly influencing the parasitism are stipulated, they may be tested on various grounds in small-scale assays and observations, in order to plan the appropriate design of the transgenic framework. In this way, cell and tissue culture models of parasitism involving trees and mistletoes may be indispensable and may generate proof(s) of concept(s). Then, a focused transgenic gene manipulation and characterization stage would involve lab-to-field-scale testing and validation of the successfully developed events. Translational activities would follow commercialization requisites on a case-by-case basis.

Research into transgenics would require a greater understanding of mistletoe’s genetics and molecular basis, as well as the dynamics and kinetics of materials’ flow between the host and its parasite (Figure 4). Alternatively, where the same mistletoe parasitizes multiple hosts, it might be interesting to identify similar genetic and/or molecular elements, their influences, and their interactions within these frameworks. In addition, a comprehensive comparison of meta-analyses arising from infection instances between the same host and different parasite pairs could provide insight into rigidities, flexibilities, crucial events, genetic elements, and/or their encoded influences within these frameworks. These characterizations may lead to the development and testing of transgenic host(s) that are resistant to mistletoe attacks, but also produce fruit and timber in a qualitative and quantitative manner. Technically speaking, genetic engineering is endowed with certain choices of gene knockout and gene silencing options. In addition to site-directed mutagenesis, RNA antisense, small interfering RNA (si-RNA), and the CRISPR-Cas9 approaches, there are time-tested ways to manipulate transcription factors [347,348,349,350,351]. The use of transgenic tree crops for translational research (Figure 5) would revolutionize and open new avenues for commercial fruit and timber businesses worldwide, eliminating the need for the cost-, time-, and labor-intensive management of mistletoe pests, and potentially ending primitive conventional methods that are not eco-friendly. Other than this, transgenic approaches may be combined with chemical control options. This may be realized by developing hosts that can withstand the otherwise mistletoe-intolerable levels of herbicides while delivering them to deter mistletoe hemiparasites. In this arena, we believe that much of the background work has already been done, as in *Oronache* and *Striga* [262,352], and can also be seen in the launch of a transcriptome meta-analysis initiative, the Parasitic Plant Genome Project [353]. There are a variety of reports that offer foundational studies on tree functional genomics [354,355,356,357,358,359]. As highlighted before, biotechnological approaches such as genetic engineering and plant tissue culture [211,360] have been underutilized, which indeed could represent a step away from conventional breeding strategies. There are few examples where tree transformation has been attempted in this regard. This should be seen as a critical requirement for introducing the usefully valued trait into specific fruit and timber tree cultivars [361]. In mitigating the mistletoes, however, such initiatives are yet to see fruitful realization and technology translations. 

### 5.8. Role of Epigenetic Signaling in Mistletoe Parasitism: Moving beyond the Genetic Basis

It is also important for scientists to determine how mistletoe parasitism is epigenetically modulated or influenced. Mistletoes exhibit epigenetic influences in their foliar appearance, mimicking their hosts [362]. We have previously reported the unusual behavior of the loranth *Dendrophthoe falcata* var. *falcata*, exhibiting two different haustorial types on different fruit tree hosts [36]. Moreover, in areas where more probable and notable hosts were present, only a few uncommon hosts succumbed to infection by *D. falcata* [36]. Why was this so? It would be imperative to test whether the epicortical roots emerging from *D. falcata* from one host can attach to a different or the same host in proximity. In a similar manner, some of the other basic questions that are intriguing in this context may be as follows: What signals enable mistletoe seeds to germinate on existing hosts? How do mistletoes expand their host tropism in light of the ever-increasing list of newer hosts? Answers to these questions would offer more useful and efficient strategies to control mistletoes. One such strategy could consider manipulating plant signaling by transgenic and epigenetic interventions, and these may easily be integrated into pest management plans such as silviculture. Both mistletoes (for infection) and their host(s) (for defense) inflict changes in in each other’s physiological and chemical profiles [53,86]. There is still uncertainty as to whether mistletoe–host interactions involve any chemical warning signals analogous to the aposematism seen in other parasites [363]. On the mistletoe’s end, some investigative leads have been gained; for example, the gelatinous seed coat material in *Phoradendron* spp., viscin, is known to signal host/non-host responses [87,88]. Furthermore, to initiate attachment with the host, the mistletoes require seed germination stimulants (such as ethylene and nucleophilic protein receptor) and a diversity of haustoria-inducing factors (HIFs) [275,364]. HIFs such as xyloglucan endotransgycosylases (XETs), expansins, glucanases, and cell wall hydrolases are expressed in mistletoes [89]. XETs may facilitate haustorial progression into hosts by delinking xyloglucans, which make up the secondary walls in the host vasculature [365,366]. Other HIFs comprise strigolactones, quinones, and flavonoids [275,367,368,369]. Oxidative degradation of host cell wall lignin by the action of peroxidases and H_2_O_2_ may also activate HIFs [370]. The defense signals to mistletoes from the host side are analogous to that against herbivores and other biotic stressors [9,108,371,372,373]. These are marked by anatomical variations such as lignification, suberization [116,374,375], and the formation of secondary growth-like wound periderm [292,376], all of which variously oppose progression processes from mistletoes [116]. In addition, hosts respond to mistletoe attack by making inherent changes to their hormone profiles, such as abscisic acid, jasmonic acid, salicylic acid, and indole-6-aminoacid [374]. This may increase levels of potent secondary metabolites such as tannins, terpenes, and phenolic compounds [9,61,109,130,332,372,373]. Furthermore, the infection site may display the accumulation of volatile compounds and ROS, in turn triggering enzyme cascades that signal for apoptosis, culminating in defoliation [61,130,374]. The signaling crosstalk involved during the mistletoe life cycle over a host should therefore be examined for potential candidates that drive the epigenetic control over chemical dynamics. It is also likely that a precise understanding of the epigenetic framework that governs mistletoe–insect interactions may facilitate easier and more effective screening for MBCAs. 

### 5.9. Smart Mistletoe Management: How the 21st Century Can Mitigate the Mistletoe Problem

**Figure 6:**
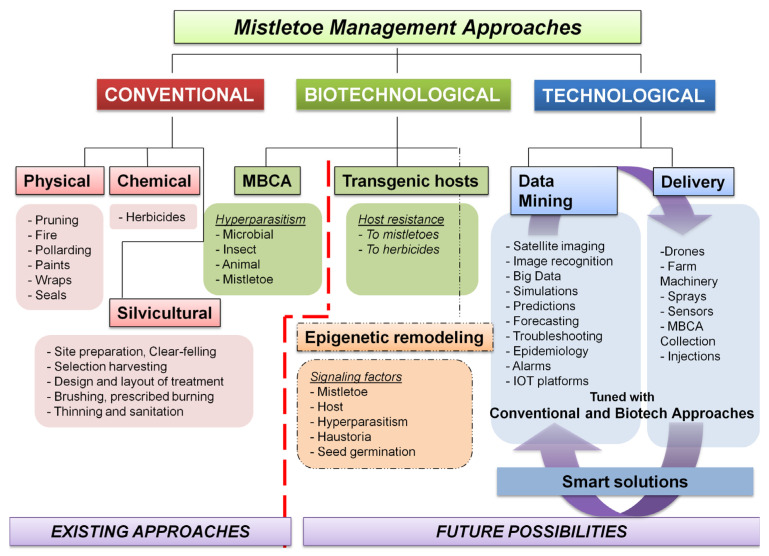
Scheme of existing mistletoe management options and future possibilities with biotechnology and technologically smart approaches. Transgenic interventions and the use of technological advancements may solve the present problems in mistletoe management. An epigenetic remodeling strategy, however, would be a far-future possibility and might be realized following a full understanding of the basis of the multitrophic interactions prevailing in the vicinity or within mistletoe–tree host associations.

The literature resources do not majorly document any new developments with conventional mistletoe management approaches due to technical glitches or technological paucity. Recently, a group described a novel handheld tool, ‘Mistletoe Eradicator’, which simultaneously provides both mechanical pruning and chemical treatment [377]. The 21st century offers a huge range of possibilities with advancements into big data, automation, artificial intelligence, and platforms such as the Internet of Things (IoTs). Newer technologies such as artificial intelligence, satellite imaging (remote sensing), image recognition, drones, and weather forecasting can be utilized in substantiating smart solutions for mistletoe pest management programs (Figure 6). For example, intelligent drones may permit the aerial screening of forest canopies for mistletoes, as well as for carrying out physical (pruning) and chemical treatments (herbicide spraying, branch injections, etc.). To make this possible, detection algorithms would need precision in screening and classifying mistletoes, which might face challenges resulting from the huge diversity of mistletoes, their growth stages, geographical settings, climates, canopy structures, size, density, etc. [86,378,379,380,381,382,383]. Moreover, many mistletoe species are known to mimic their hosts in foliar structures [139]. Moser and Campione showed the successful use of Google Earth as a remote sensing tool to detect eastern spruce dwarf mistletoe in the forests of Minnesota [384]. A group from Mexico recently developed a genetic programming-based algorithm that screens mistletoe *Phoradendron velutinum* using multispectral aerial images collected on a radiation sensor fitted to an unmanned aerial vehicle [385]. This algorithm classifies *P. velutinum* based on its flowering stage. Before this, several groups had developed other algorithms based on thermal imaging, hyperspectral lines, convolutional neural networks, canopy height, colorimetry, etc. [386,387,388,389,390,391,392]. If developments into these dimensions are studied further, the effective selection of conventional and biotechnological treatment approaches may lead to a productive synchrony with silviculture. Silvicultural decisions such as treatment area and treatment schedule depend on precise knowledge of and attention to the epidemiology of mistletoes and their pathogens, mistletoe population dynamics, and the silvics of the mistletoes’ hosts [34]. In this regard, even automation and artificial intelligence solutions may offer better time-saving, labor-saving, and cost-saving prospects, as well as being amenable to modifications on a case-by-case basis. 

## 6. Conclusions

Mistletoes have undoubtedly acquired pest status in many countries [26]. By drawing resources from other plants in communities with higher trophic levels, they parallel herbivores [108]. They are viewed as robbers by fruit and timber farmers as they present a constant problem as parasitic plants in commercial plantations. In spite of their inconsistency, cost and labor-intensiveness, a lack of technological advancements in terms of delivery systems, the impact on nearby plants, ecological disturbances, and negative environmental effects, physical and chemical control approaches have been somewhat utilized in attempting to manage this concern and have proven ineffective, if not failed. Integrated management programs, though deemed relatively effective, are practiced only on small scales and in a few countries [262]. Despite the fact that many organismic entities have been discovered to be detrimental to mistletoes, exploring and exploiting their potential as successful MBCAs has not been well realized to date. Biocontrol strategies can be synchronized with silvicultural approaches; given that models where MBCA treatment options, schedules, and mistletoe life cycles are available, this could help in framing preferred plantation plans. Other than this, where MBCAs are not a practicable solution, technological advancements are highly sought to resolve drawbacks with physical and chemical treatment methods. During our discussion, we explored the smart interventions and big data trends of 21st-century technological advancements. These ideas and opinions hold strong potential for the combination of conventional and bio/technological approaches (Figure 6). Control strategies must take into account the biological interactions and dynamics among community structures, which maintain the niche’s ecological propensities.

Considering the aforementioned promising approaches, and not neglecting the ecological value of the mistletoes (in being a keystone resource for biodiversity), the genetic engineering of hosts to develop, induce, and enhance specific resistance will be greatly rewarding. Even though its benefits include being environmentally safe and with no use of chemical agents, due to the additional labor, costs, complicated management, as well as potential to wipe out the parasite seed banks on hosts, this approach has remained unrealized [262]. Such transgenic trees would satisfy both (i) the conservation of the biodiversity and medicinal value aspects of mistletoes and (ii) aims to foresee a fruit and timber business that is unaffected by mistletoe. Successful examples of the transgenic approach are known in relation to other parasitic weeds, such as *Striga*, *Orobanche*, and *Cuscuta* [393,394,395,396]. In this regard, in this study, we intended to supply blueprints for transgenic and translational research into developing and commercializing stable mistletoe-resistant tree cultivars.

In this review, the authors do not underestimate the ecological significance and medicinal value of mistletoes, both in traditional and modern treatment practices. However, this article emphasizes the development and implementation of more feasible management solutions for highly damaging mistletoes that affect commercial tree plantations. The risks surely outweigh the usefulness of mistletoes in such settings, as others pointed out long ago [230]. Thus, tree decline, combined with mistletoe, cannot be overlooked in itself as a major factor negatively impacting biodiversity and commerce. Hopefully, the biotechnological and smart management approaches discussed here, if operationalized in the future, should serve as a paradigm shift in mistletoe management.

## Data Availability

Not applicable.

## Supplementary Materials

The following supporting information can be downloaded at: https://www.mdpi.com/article/10.3390/biology11111645/s1, Table S1: List of potential MBCAs yet known for some mistletoe genera. References [397,398,399,400,401,402,403,404,405,406,407,408,409,410,411,412,413,414,415,416] are cited in the supplementary materials.
