# Supplementary material for: Mitigating the Mistletoe Menace: Biotechnological and Smart Management Approaches
Source: Biology (Basel). 2022 Nov 10;11(11):1645. doi: 10.3390/biology11111645 (PMC9687506; doi:10.3390/biology11111645)
Supplement: Supplementary file 1 [file biology-11-01645-s001.zip › biology-1988167-supplementary.pdf]

Supplementary Information

Potential MBCA candidates	Entity type/ common name	Features and possibilities as MBCA	Mistletoe target(s)	Mistletoe host(s)	Refs.
***Acremonium kiliense* Grütz**	Microbe: Fungi	Strong disease symptoms	*Viscum album*	Crimean pine (*Pinus nigra*)	[217]
***Alternaria alternata* (Fr.) Keissl.**	Microbe: Fungi	Strong disease symptoms	*Viscum album*	Crimean pine (*Pinus nigra*)	[217]
Anarsia sagmatica* Meyr *[Lep.: Gelechiidae]	Insect: fruit fly	Larvae feeding on all foliar parts to completely destroy the host, well synchronized with flowering period of host, multivoltine having up to 4 generations a year.	*Dendrophthoe falcata*. L.f Ettingsh	As above	[231]
Apion variegatum* Wenck *[Apio: Apioninae]	*Insect: Weevil*	Can make holes in the stems	*Yellow berried mistletoe (Loranthus europaes), V. album*	Fir tree (*Abies alba Mill.*) sessile oak (*Quercus petraea* /Matt./ Liebl.), pedunculate oak (*Q. robur* L.), narrow-leaved ash (*Fraxinus angustifolia* Vahl), common locust (*Robinia pseudoacacia* L.), sycamore (*Acer pseudoplatanus* L.) and black alder (*Alnus glutinosa* /L./ Gaertn.).	[144] [397,398]
Bacillus megaterium	Microbe: Bacteria	Showed pathogenic symptoms when injected into mistletoe, but not when sprayed.	*Viscum album*	Crimean pine (*Pinus nigra*)	[217]
Bacillus pumilus	Microbe: Bacteria	Showed pathogenic symptoms when injected into mistletoe, but not when sprayed.	*Viscum album*	Crimean pine (*Pinus nigra*)	[217]
Burkholderia cepacia	Microbe: Bacteria	Showed pathogenic symptoms when injected into mistletoe, but not when sprayed.	*Viscum album*	Crimean pine (*Pinus nigra*)	[217]
Cacopsylla visci* Curtis,1835 *[Hemi:Psyllidae]	Insect: British bug, jumping plant louse	Monophagous on mistletoe, sap sucking	*Yellow berried mistletoe (Loranthus europaes), V. album*	Fir tree (*Abies alba Mill.*) sessile oak (*Quercus petraea* /Matt./ Liebl.), pedunculate oak (*Q. robur* L.), narrow-leaved ash	[144, 397—399]

				(*Fraxinus angustifolia* Vahl), common locust (*Robinia pseudoacacia* L.), sycamore (*Acer pseudoplatanus* L.) and black alder (*Alnus glutinosa* /L./ Gaertn.).	
***Caliciopsis arceuthobii* (formerly *Wallrothiella arceuthobii*)**	Microbe: Fungi	Attacks spring-flowering mistletoes, destroys fruits and seeds, can grow in vitro, potential is limited by large, annual variations of infection.	*Arceuthobium pusillum, A. americanum, A. douglasii,* and *A. vaginatum*	Western hemlock (*Tsuga heterophylla*), lodgepole pine (*Pinus contorta*)	[197, 198, 255, 400]
Carulaspis visci* Schrank, 1781 *[Dias: Carulaspis]	Insect: Scale	Exclusively occurs on mistletoe, causes galls on leaf.	*Yellow berried mistletoe (Loranthus europaes), V. album*	Fir tree (*Abies alba Mill.*) sessile oak (*Quercus petraea* /Matt./ Liebl.), pedunculate oak (*Q. robur* L.), narrow-leaved ash (*Fraxinus angustifolia* Vahl), common locust (*Robinia pseudoacacia* L.), sycamore (*Acer pseudoplatanus* L.) and black alder (*Alnus glutinosa* /L./ Gaertn.).	[144] [397,398]
Celypha woodiana* Barret, 1882 *[Tort: Olethreutina]	Insect: Mistletoe Marble moth	Threatened insect, needs conservation as priority as for MBCA assessments.	*Viscum album*	Apple	[238]
Ceratitella asiatica* Hardy *[Dipt.: Tephritidae]	Insect: fruit fly	Larvae Feeds on fruits, no natural enemies known, 2 generations a year.	*Dendrophthoe falcata* L.f Ettingsh	As above	[231]
***Colletotrichum gloeosporioides* (Sacc.) Penz.**	Microbe: Fungi	Not host specific, appear as small, brown to black, necrotic lesions on the nodes of fruits and shoots, not recovered from endophytic system, easy, inexpensive culturing, germinates over a wide temperature range, disrupts development of mistletoe shoots, preventing reproduction. As attacks any time after shoot emergence, so flexible application scheduling.	*Arceuthobium spp., Dendrophthoe falcata* L.f Ettingsh	Western hemlock, Lodepole pine	[401—406]
Cylindrocarpon cylindroides	Microbe: Fungi	Destroys the endophytic system	Dwarf Mistletoe *Arceuthobium tsugense*	Western hemlock	[211, 407]
Cylindrocarpon gillii	Microbe: Fungi	Causes anthracnose in shoots, needs climate matching as inconclusive	Dwarf mistletoes *Arceuthobium americanum,*	Western hemlock, Lodepole pine	[197, 405]

		results (from inoculating an isolate of a warm, dry climate to a cool, moist condition)	*A. douglasii*, *A. tsugense* subsp. *tsugense*		
Cytospora abietis Sacc.	Microbe: fungi	Kills mistletoe-infected branches, infectivity needs assessment as occasionally also parasitizes non-mistletoe-infected branches.	*Arceuthobium abietinum*	*Abies magnifica* A.Murr. and *Abies concolor* (Gord. & Glend.) Lindl.	[408]
Delias eucharis* Dr. *[Lep.: Pieridae]	Insect: butterfly	Larvae Feeding on leaves, no alternate host.	*Dendrophthoe falcata* L.f Ettingsh, *Loranthus cordifolius*	As above	[231]
Demarchus pubipennis* Jacoby *[Col.: Chrysomelidae]	Insect: beetle	Larvae Feeding on leaves, heavily damages host leaves, multivoltine having up to 4 generations a year.	*Dendrophthoe falcata* L.f Ettingsh	As above	[231]
Euzopherodes ephestialis* Hamps *[Lep.: Pyralidae]	Insect: snout moth	Larvae feeds on flowers, multivoltine with 5-6 generations a year.	*Dendrophthoe falcata* L.f Ettingsh, *Arceuthobium oxycedri* (DC.) M. Bieb., *Korthalsella opuntia* (Thunb.) Merrill., *Viscum album* L.	*Acacia modesta* [22]	[231]
***Gibberidea visci* (Fuckel)**	Microbe: fungi	Facultative fungi	*Viscum album*	Crimean pine (*Pinus nigra*)	[409]
Icerya purchase* (Maskell) *[Hemi.: Monophlebidae]	Insect: cottony-cushion scale	Causes heavy infection on mistletoe, however MCBA potential is limited due to known off target infection possibly on citrus and other trees	*Viscum cruciatum* (red berried mistletoe)	Olive tree (*Olea europaea*)	[240]
Ixapion variegatum* Wencker. 1864 *[Apio: Apioninae]	Insect: mistletoe beetle	Lays eggs in mistletoe stem, kills buds	*Viscum album*	Apple	[238, 410]
Mylothris sagala* Grose-Smith, 1886 *[Lep.: Pieridae]	Insect: Butterfly, dusky dotted border	Feeds on sap, oviposits on mistletoe	*Yellow berried mistletoe (Loranthus europaes), V. album, Loranthus freisiorum*	Fir tree (*Abies alba Mill.*) sessile oak (*Quercus petraea* /Matt./ Liebl.), pedunculate oak (*Q. robur* L.), narrow-leaved ash (*Fraxinus angustifolia* Vahl), common locust (*Robinia pseudoacacia* L.), sycamore (*Acer pseudoplatanus* L.) and black alder (*Alnus glutinosa* /L./ Gaertn.).	[144] [397, 398]
Myrmex arizonicus [Col.: Curculionidae: Conoderinae]	Insect: Ant-like Stem borer weevil	Can make round exit holes in diameter in the stems, with larval galleries, can kill mistletoe shoots	*P. villosum, Phoradendron on oak (Santa Cruz County), P. flavescens (Pursh.) Nutt., P.*	Oak, *Quercus lobata, Juglans major, Juniperus sp., Prosopis sp.*	[226]

			juniperinum Engelm., *P. tomentosum*		
Myrmex sp.* *[Col.: Curculionidae: Conoderinae]	Insect: Stem borer weevil	Can make round exit holes about 2 mm in diameter in the stems, with larval galleries, can kill mistletoe shoots	*Phoradendron serotinum*	Water oak (*Quercus lobata*)	[225]
Neonectria neomacrospora	Microbe: Fungi	Selective to mistletoe infected host branches, invades host without wounding, proven pathogenicity, host girdling, reduces mistletoe shoot growth, rapid cankering, abundant sporulation, awaits MBCA formulation and delivery technology improvement.	*Arceuthobium tsugense, A. occidentale*	Western hemlock, *Pinus miricata* D.Don	[213, 405, 410]
Pandoraea pulminicola	Microbe: bacteria	Showed pathogenic symptoms when injected into mistletoe, but not when sprayed.	*Viscum album*	Crimean pine (*Pinus nigra*)	[217]
***Phaeobotryosphaeria visci* Arx & E. Mull. [Syn.: *Botryosphaeria visci* (Kalchbr.)]**	Microbe: Fungi	Induces chlorotic symptoms on leaves, complete destruction of mistletoe.	*Viscum album*	*Populus nigra, Acer saccharunum, A. pseudoplatanus*	[129, 411, 412]
Pinalitus viscicola* Pumton, 1988 [syn: *Orthops viscicola, Lygus viscicola*] *[Hemi:MIridae]	Insect: plant bug	Monophagous on mistletoe, sap sucking	*Viscum album*	Apple	[238, 399]
Pseudaulacaspis cockerelli (Cooley) [Hemi.: Diaspididae]	Insect: False oleander scales, Scale insect	Infection on leaf and haustorial structures, no infection on host, higher density of flakes on mid-rib and veins in leaves. Requires further evaluation.	*Dendrophthoe falcata* L.f Ettingsh	Cassia tree (*Senna siamea*)	[37]
***Sphaeropsis visci* Fr.**	Microbe: fungi	Facultative, found on mistletoe leaves and fruits	*Phoradendron spp., Dendrophthoe falcata* L.f Ettingsh, *Viscum coloratum*	Various trees	[413-416]
Stugeta bowkeri* Trimen, 1864 *[Lep.: Lycaenidae]	Insect: Butterfly, Bowker's Sapphire	Feeds on sap, oviposits on mistletoe	*Yellow berried mistletoe (Loranthus europaes), Viscum album, Viscum rotundifoliumm Loranthus elegans* and *L. oleaefolius*	Fir tree (*Abies alba Mill.*) sessile oak (*Quercus petraea* /Matt./ Liebl.), pedunculate oak (*Q. robur* L.), narrow-leaved ash (*Fraxinus angustifolia* Vahl), common locust (*Robinia pseudoacacia* L.), sycamore (*Acer*	[144, 397, 398]

				pseudoplatanus L.) and black alder (*Alnus glutinosa* /L./ Gaertn.).	
Synanthedon loranthi **Kralicek, 1966** ***[Lep.: Sesiidae]***	Insect: Moth	Sap feeding larvae.	*Yellow berried mistletoe (Loranthus europaes), V. album, Viscum album abietis, Viscum album austriacum, Viscum laxum* and *Loranthus europaeus*	As above and Scot pine.	[144,397-398]
Timorus sarcophagoides ***[Col.: Curculionidae: Conoderinae]***	Insect: Weevil	Lives exclusively on the mistletoe, exhibits evasive mimicry (potentially consistent efficacy as MBCA), pieces holes into mistletoe endophytic system during oviposition, life cycle matches with mistletoe flowering peak.	Parrot flower mistletoe (*Psittacanthus robustus*)	*Not available*	[229, 228]
Zelleria loranthivora **Meyr.** ***[Lep.: Yponomeutidae]***	Insect: moth	Larvae destroy flowers by feeding and boring, no alternate host known.	*Dendrophthoe falcata* L.f Ettingsh	As above	[231]

Table S1. List of potential MBCAs yet known for some mistletoe genera. Note: *Dendrophthoe falcata* was formerly known as *Loranthus longiflorus.* Also, *Lep.: Lepidoptera, Dipt.: Diptera; Col.: Coleoptera,* Tort.:*Tortricidae, Apion: Apionidae.*
